# Multifunctional MeHA hydrogel for living materials delivery with enhanced cartilage regeneration

**DOI:** 10.3389/fbioe.2025.1545773

**Published:** 2025-05-30

**Authors:** Qunchao Chen, Lang Bai, Guoyang Wan, Yuefeng Hao, Xing Yang, Hongtao Zhang

**Affiliations:** ^1^ Department of Orthopedics, The Fourth Affiliated Hospital of Soochow University, Suzhou Dushu Lake Hospital, Medical Centre of Soochow University, Suzhou, Jiangsu, China; ^2^ Orthopedics and Sports Medicine Center, The Affiliated Suzhou Hospital of Nanjing Medical University, Suzhou Municipal Hospital, Gusu School, Nanjing Medical University, Suzhou, China

**Keywords:** particulated juvenile articular cartilage, living materials, lubrication, cartilage repair, MeHA

## Abstract

Particulated juvenile articular cartilage (PJAC) has emerged as a promising living material for articular defect treatment. However, the fragile nature of PJAC hinders its wide clinical application. Here, inspired by the chemical composition and hierarchical structure of natural cartilage, we developed a novel hydrogel carrier system for PJAC delivery. Our carrier system, MeHA@J@DM, utilized methacrylated hyaluronic acid (MeHA) to incorporate PJAC and coated it with a polymerized mixture of dopamine methacrylamide (DMA) and 2-methylacryloyloxyethyl phosphorylcholine (MPC), forming an adhesive lubricant, p(DMA-MPC). MeHA@J@DM exhibited excellent performance for PJAC protection with enhanced cell viability, bioactivity, and lubrication properties. We evaluated the effectiveness of MeHA@J@DM in cartilage cell migration, where juvenile cartilage showed greater efficiency and remodeling abilities. *In vivo* rabbit cartilage defect models demonstrated superior cartilage regeneration with the MeHA@J@DM hydrogel. Our findings suggest that MeHA@J@DM has translational potential for PJAC implantation to enhance cartilage regeneration and benefit patients with articular cartilage lesions.

## 1 Introduction

Articular cartilage lesions resulting from acute injury, trauma and progressive degeneration could lead to severe pain, functional disability and physical activity restriction of the joints (Nordberg et al., 2022). Various strategies are currently employed for cartilage repair. Reparative techniques, such as marrow stimulation procedures (e.g., drilling, microfracture, and abrasion arthroplasty), offer arthroscopic solutions without donor site morbidity (Redondo et al., 2018; Gudas et al., 2006). These techniques promote the formation of fibrous repair tissue through deliberate subchondral bone penetration, leading to satisfactory clinical outcomes in a significant number of patients. However, the biomechanical characteristics of fibrocartilage are notably inferior to those of native hyaline cartilage, which primarily comprises type II collagen (Jagtenberg et al., 2021). Restorative techniques, such as cartilage autograft transfer, have demonstrated subjective improvements in pain relief and functional restoration. Cartilage autograft transfer entails the relocation of viable chondrocytes within the native extracellular matrix and subchondral bone from minimally weight-bearing areas of the knee joint (Lavender et al., 2020). While these strategies have demonstrated success to some extent, there are limitations and challenges associated with each approach. For example, cartilage autograft transfer requires harvesting healthy cartilage from another site, which may lead to donor site morbidity (Krych et al., 2012).

Particulated juvenile articular cartilage (PJAC) composed of minced hyaline cartilage derived from young donors has emerged as a novel living material for the treatment of symptomatic articular defects (Changgui et al., 2020). The PJAC has presented fascinating cartilage regeneration capacity with increased chondrocyte population and higher metabolic activity (Ao et al., 2019). Notably, some pioneer studies have demonstrated the superior translational potential of juvenile chondrocytes in generating hyaline-like cartilage tissue compared to older counterparts. Nevertheless, the PJAC implantation is still far from wide clinical application due to the fragile nature of the PJAC (Lavender et al., 2020; Stevens et al., 2014). For example, direct PJAC implantation is always accompanied with compromised cell viability and bioactivity due to detrimental inflammatory microenvironment after the cartilage lesions and the unavoidable contamination after implantation (Li et al., 2022). More importantly, the inevitable friction between two articular surfaces could further induce excess shear force to decrease the cell viability and further provoke exorbitant inflammation, which may cause the failure of the cartilage autograft grafts (Espinosa et al., 2021). Thus, the development of novel carriers for PJAC delivery is of great clinical relevance to enhance cartilage regeneration.

**FIGURE 1:**
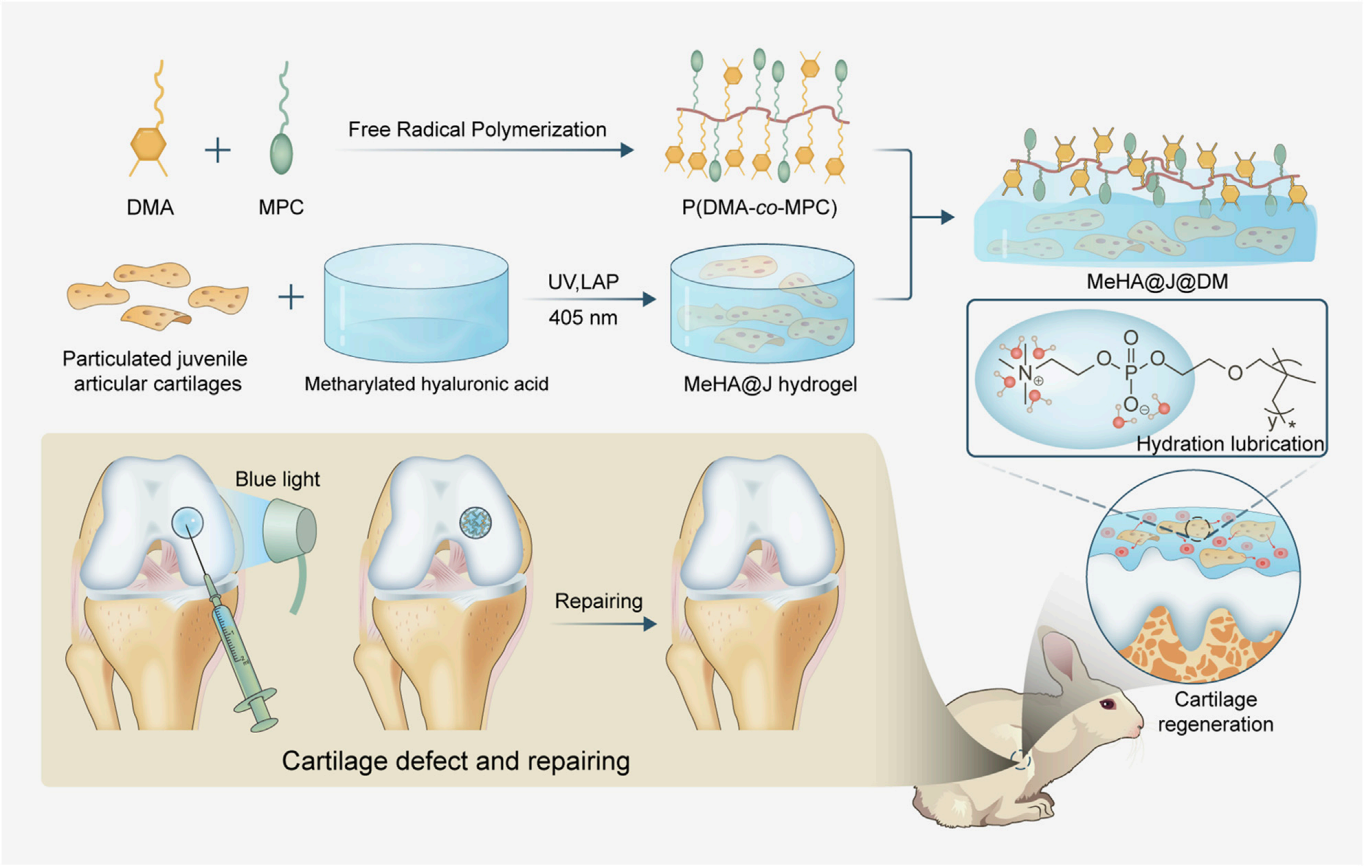
Schematic of the natural cartilage inspired carrier for living materials delivery with enhanced cartilage regeneration. The MeHA worked as the basal carrier materials and the adhesive lubricant p(DMA-MPC) could mimic the natural hydration layer of the cartilage surface with powerful lubrication. MeHA@J@DM could alleviate the inflammatory microenvironment to further boost the chondrogenesis of the cells and present excellent lubrication under physiological conditions and tremendous anti-contamination capacity to foulant and bacteria to boost cartilage regeneration.

Here, inspired by the chemical composition and hierarchical structure of natural cartilage, we have developed a novel hydrogel carrier system for effective living materials delivery such as PJAC with enhanced articular cartilage regeneration (Figure 1). Firstly, we synthesized the methacrylated hyaluronic acid (MeHA) as the basal carrier materials. In nature, HA is the main chemical composition of cartilage with superior biocompatibility, intrinsic regenerative capacity and anti-inflammation potential to support chondrocytes growth and cartilage regeneration (Ondeck and Engler, 2016; Xu et al., 2022; Lei et al., 2022). Then, we co-polymerized the dopamine methacrylamide (DMA) and the zwitterionic 2-methylacryloyloxyethyl phosphorylcholine (MPC) to obtain the adhesive lubricant p(DMA-MPC), which could mimic the natural hydration layer of the cartilage surface with powerful lubrication (Liu et al., 2019). We next coated the p(DMA-MPC) onto the PJAC encapsulated MeHA (MeHA@J) to construct the final delivery system (MeHA@J@DM). We found the carrier system (MeHA@J@DM) exhibited excellent performance for cell encapsulation with enhanced cell viability and bioactivity. Meanwhile, our natural cartilage delivery system was further evaluated for its effectiveness in cartilage cell migration, comparing juvenile and adult cartilage. Juvenile cartilage demonstrated sustained high efficiency in promoting cartilage cells’ migration. This finding holds promise for the reparative filling of cartilage defects, as juvenile cartilage cells exhibited greater expression of MMP-14 compared to adult cartilage. This suggests that juvenile cartilage-derived cells possess enhanced remodeling abilities. Moreover, we found the MeHA@J@DM with the hierarchical hydration layer presented excellent lubrication under physiological conditions. The *in vivo* rabbit cartilage defect model further demonstrated the enhanced cartilage regeneration of our MeHA@J@DM hydrogel. In all, we believe our MeHA@J@DM demonstrated excellent translational merits for PJAC implantation to boost cartilage regeneration and benefit patients suffering from articular cartilage lesions.

## 2 Materials and methods

HA (MW = 200 kDa) were purchased from Macklin (Guangzhou, China). All antibodies were purchased from Abcam (Cambridge, MA, United States). Unless otherwise specified, all other reagents were purchased from Aladdin (Shanghai, China).

### 2.1 Synthesis and characterization of MeHA and DMA-MPC

The MeHA was prepared following a previously described method (Tsanaktsidou et al., 2019). Typically, a mixture of 100 mL of 2% HA and 10 mL of methacrylic anhydride (Macklin, China) was adjusted to a pH of 8 using NaOH solution (Macklin, China). After keeping the mixture of methacrylic anhydride and HA at 4°C for 2 days, the initial MeHA product was precipitated and washed with ethanol five times. Finally, the precipitated MeHA was collected and subjected to vacuum freeze-drying. The resulting particles were modified with MPC, as described in a previous study. In brief, a solution containing 0.1 g of DMA (Macklin, China) and 0.6 g of MPC (Macklin, China) dissolved in 25 mL of N, N-dimethylformamide (Macklin, China), which also contained 1% azodiisobutyronitrile (Macklin, China), was reacted at 65°C for 36 h. The reaction products were then collected and subjected to dialysis for 48 h to obtain DMA-MPC. The successful synesis of MeHA and DMA-CO-PMC were characterized by ^1^H nuclear magnetic resonance and (Bruker, ^1^H NMR, German) and fourier-transform infrared spectroscopy (FTIR, Thermo Fisher, China).

### 2.2 Preparation of the PJAC and MeHA@J@DM

Particulated juvenile articular cartilages (PJAC) were obtained from juvenile New Zealand rabbits (∼1.0 kg, 6–8 weeks) procured from Zhaoyan (Suzhou) New Drug Research Center Co., Ltd. Following euthanasia, aseptic techniques were employed to open the knee joints and expose the cartilage on the femoral condyles. Subsequently, cartilage slices were separated using a scraping knife and rinsed with DMEM/F12 medium (Gibco, China). The cartilage was then meticulously prepared into fragments measuring approximately 3 mm in length, width, and 0.5 mm in thickness using ophthalmic scissors (Riboh et al., 2015). For the subsequent experiments, a 3 w/v% mixture of MeHA precure with 0.1 w/v% LAP (EFL, China) as the photoinitiator was prepared. MeHA@J was prepared by mixing PJAC with the MeHA solution at a concentration of 20 w/w% followed with blue light irradiation for 2 min. Then, MeHA@J@DM was obtained by simply dipping 50 µL p(DMA-MPC) solution (2 mg/mL in water) onto the surface of crosslinked MeHA@J.

### 2.3 Physical characterization of MeHA@J@DM

Different samples (MeHA, MeHA@J and MeHA@J@DM) were prepared following the methods mentioned before. After freezing dried, the micromorphology of the hydrogels was observed by scanning electron microscope (Hitachi, Japan) (Kim and Chu, 2000). To evaluate the lubrication performance of MeHA@J@DM, a universal material testing machine (UMT-3, Bruker Nano Inc., Germany) was employed. The testing setup involved fixing the hydrogels within polyethylene (PE) models, with an 8-mm-diameter PE ball serving as the upper specimen. During the test, the time coefficient of friction (COF) curve was recorded (Han et al., 2022). The mechanical properties of the hydrogels were tested by a universal mechanical equipment (Instron, United States). The compressive speed is 1 mm/min at room temperature (Xu et al., 2023).

### 2.4 Biocompatibility evaluation of MeHA@J@DM

The biocompatibility of MeHA@J@DM was evaluated by incubating it for 3 and 14 days. The cell culture medium utilized in the experiment was prepared by adding 10% fetal bovine serum (FBS) and 1% penicillin/streptomycin (PS) to DMEM/F12 medium (Gibco, China). Cell viability and proliferation were analyzed at 3 and 7 days after incubation using the Live/Dead kit (Thermo Fisher, China) and CCK8 quantification assay (Biosharp, China). Cell viability was determined by calculating the proportion of viable cells (green fluorescence) to the total number of cells in six randomly selected images (Zhang et al., 2022). H&E staining was performed according to the manufacturer’s instructions to observe the morphology of the cartilage cells. Cell proliferation was assessed after 3 and 7 days of culture using the CCK8 assay measure by a plate-reader with absorbance at 450 nm (Tao et al., 2020).

### 2.5 Cell migration assay of MeHA@J@DM

To further compare the migration of chondrocytes in particulated cartilage, we utilized adult rabbit particulated cartilage (MeHA@A@DM) as a control group and extended the duration of *in vitro* culture to 14–21 days. Initially, we conducted cell culture and performed live/dead staining in cell culture plates using the same methodology as described before. Furthermore, we seeded the hydrogel-cartilage composites into transwell culture plates to evaluate the vertical migration of the cartilage cells (Leijs et al., 2017). To evaluate cell migration in the transwell system, the hydrogel-cartilage composites were placed in the upper chambers of the transwell plates, while the lower chambers contained the culture medium. Following the designated incubation periods for 7, 14, and 21 days, the migrated cells were stained with crystal violet and visualized using microscopy (Nikon, Japan). The number of migrated cells in the transwell chambers was quantified using ImageJ software (NIH, United States).

### 2.6 MMP-14 expression of juvenile articular cartilages

After culturing the hydrogel-cartilage composite constructs from different groups for 7 days, we performed the MMP-14 immunofluorescent of the cultured cartilages (Yang R. et al., 2021). Briefly, the culture medium was aspirated, and the constructs were washed twice with PBS. Subsequently, the constructs were fixed with 4% paraformaldehyde for 30 min. The paraformaldehyde was then removed, and the constructs were washed twice with PBS. To enhance immunostaining permeability, Triton X-100 was added and incubated for 10 min on ice. Next, blocking buffer was added and incubated for 1 h. After removing the blocking buffer, the constructs were washed twice with PBS. Anti-MMP-14 antibody (1:200, Affinity Bioscience, China) was added and incubated overnight at 4°C. After washed by PBS, goat anti-rabbit IgG 647 (1:200, Affinity Bioscience, China) and FITC-phalloidin staining solution (1:1,000, Yeasen, China) were added and incubated for 1 h at 37°C. Finally, DAPI solution (1:2,000, Yeasen, China) was added and incubated for 10 min at 37°C. Confocal laser scanning microscope (Zeiss, German) was used to observe the expression of MMP-14. The fluorescence intensity of the images was quantified using ImageJ software. We also collect the cell culture medium from MeHA@J@DM and MeHA@A@DM and then performed the MMP-14 ELISA test (Abcam, United Kingdom) (Kasurinen et al., 2018). The concentrations were calculated according to the standard curve.

### 2.7 *In vivo* biological characterizations of the MeHA@J@DM

Approval for all animal evaluations was obtained from the Ethics Committee of the Affiliated Suzhou Hospital of Nanjing Medical University (Approval Number: K-2023-044). A total of 24 New Zealand white rabbits, weighing between 2.0 and 3.0 kg and male, were randomly divided into four groups: blank group, MeHA group, MeHA@J group, and MeHA@J@DM group. We used ketamine (20 mg/kg) into rabbits via intravenous injection method for anesthesia. A dental drill was used to create a cartilage defect with a diameter of 5 mm and a depth of 0.3 mm in the trochlear groove of the rabbits’ distal femur. The defects were then filled with the respective hydrogels and exposed to UV irradiation for 2 min. The knee joints were closed with 4–0 sutures, and intramuscular antibiotics were administered. After a 12-week implantation period, the rabbits were euthanized using CO_2_ suffocation, and femur samples were collected. The quality of osteochondral repair was assessed by two trained and blinded observers using the ICRS scoring system (Numata et al., 2019). Histological analysis, including H&E staining and Safranin O staining, was performed to evaluate cartilage repair at the defect sites. The histological repair score was calculated using a modified method based on previous studies (Orth et al., 2012).

### 2.8 H&E staining

Soak the slices in xylene for 15 min, then repeat again, and finally in a combination of xylene and anhydrous ethanol for 5 min. Then, apply 100%, 90%, 80%, and 70% ethanol gradients for 5 min each. Then soak for 5 min in hematoxylin staining solution, rinse with clean water, and dry. Stain with eosin solution for a few minutes, then dehydrate with ethanol gradients and inspect the slices after sealing.

### 2.9 Saffinin-O/fast green staining

Soak the slices in solid green staining solution for 2 min, then rinse with clean water. Finally, soak them in saffron coloring solution for 20 s. Use an ethanol gradient to quickly dry the slices and then inspect them.

### 2.10 Col II immunohistochemical staining

The slices were dewaxed with xylene and then coated with ethanol, as previously described. After cleaning, the slides were immersed in a 0.3% hydrogen peroxide solution for 10 min. After washing in PBS, the slides were blocked with a solution for 5 min. The diluted anti-Collagen II antibody was then incubated for 2 h at room temperature. After washing with PBS, the secondary antibody was incubated for 20 min. The slides were then immersed in a DAB developing solution for 10 min. Following washing, the slides were sealed and examined.

### 2.11 Statistical analysis

All experiments were conducted in quadruplicate, unless stated otherwise, and the results were expressed as the mean ± standard deviation. Statistical analysis was performed using GraphPad Prism Software (GraphPad Software Inc.) with Student’s t-test, one-way or two-way ANOVA, followed by Tukey’s *post hoc* test for multiple comparisons. Statistical significance was defined as *p < 0.05, **p < 0.01, and ***p < 0.001.

## 3 Results and discussion

**FIGURE 2:**
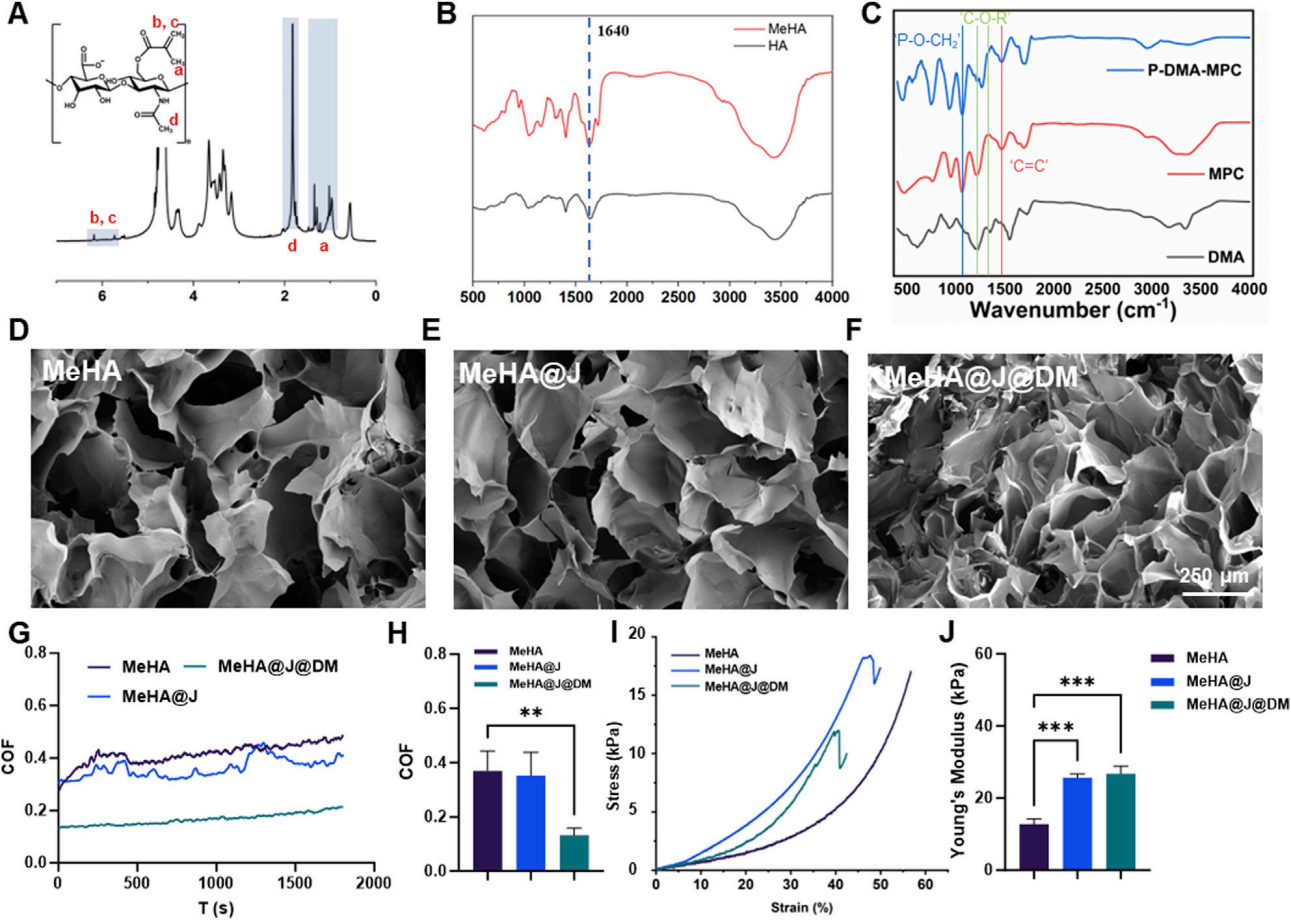
Characterization of the MeHA, DMA-MPC and MeHA@J@DM. **(A,B)** ^1^H NMR and FTIR results of the MeHA; **(C)** FTIR of the DMC; **(D–F)** The SEM images of MeHA, MeHA@J and MeHA@J@DM hydrogels. **(G–J)** COF and comprehensive mechanical properties results of the MeHA, MeHA@J and MeHA@J@DM. A difference at **p < 0.01 and ***p < 0.001 was considered statistically significant.

To formulate the MeHA@J@DM, we first synthesized the MeHA following the previous protocols. We used the ^1^H nuclear magnetic resonance (^1^H NMR) and Fourier-transform infrared spectroscopy (FTIR) to evaluate the synthesized MeHA. The peaks at ∼5.8 and ∼6.8 ppm representing the C=CH_2_ groups indicating the successful synthesis of the MeHA (Figure 2A; D'Amora et al., 2018). Additionally, FTIR evaluation with the characteristic peaks on 1,640 cm^−1^ representing the C=C groups which were in consistent with literature (Figure 2B; D'Amora et al., 2018). To bestow the MeHA@J@DM carrier with biomimicking lubrication capacity, we also synthesized the p(DMA-MPC) with the copolymerization of the adhesive DMA and zwitterionic MPC. The success synthesis of the p(DMA-MPC) was also confirmed by the FTIR evaluation with the characteristic peaks on 1,602 cm^−1^ representing the C=C groups of the MPC (Figure 2C). The peaks observed at 1,454 cm^−1^ and 1,247 cm^−1^ in p(DMA-MPC) were attributed to C-O-R (where R represents the lateral chain of MPC) and P=O stretching vibrations, respectively. Additionally, the peak observed at 1,086 cm^−1^ was assigned to P-O-CH_2_ stretching vibrations (Liu et al., 2019). After we prepared the MeHA solution with water (3 w/v %), we found the MeHA solution presented excellent flowability indicating its readily injectability during application. As expected, after crosslinking the MeHA solution with blue light for 150 s, the MeHA hydrogel could be formed demonstrating its rapid implementation capacity during critical surgeries (Chen et al., 2024). We have also validated the injectability and crosslinkability of MeHA simulating the real condition. We found the MeHA hydrogel with different structures could be constructed (Supplementary Figure S1).

Then, we mixed the prepared PJAC with the MeHA solution (20% w/v) and crosslinked the composite material with blue light for 2 min to form the PJAC loaded MeHA samples (MeHA@J). Furthermore, we coated the MeHA@J with p(DMA-MPC) with simple dipping process to prepare the final MeHA@J@DM samples. The macroscopic images of the samples clearly demonstrated the loaded PJAC in both groups (Supplementary Figure S2). For the PJAC delivery vehicles, the porous structure is of great significance to maintain the cell viability and removes the waste; thus, we used scanning electron microscopy (SEM) to evaluate the microstructure of the two groups. As expected, we find that the MeHA@J group presented the porous microstructure. Notably, the p(DMA-MPC) coated MeHA@J@DM sample also showed porous microstructure indicating that the p(DMA-MPC) coating wound not affect the porous microstructure of the MeHA hydrogel (Figures 2D–F; Han et al., 2021).

For the successful implantation of the PJAC loaded hydrogel, the interfacial lubrication is critical which could reduce the shear forces imposed on the PJAC after implementation to synergistically increase the cell viability. Therefore, we used the universal materials tester to validate the lubrication efficiency of our developed MeHA@J@DM carrier. We find the MeHA and MeHA@J groups presented around 3.5 coefficient of friction (COF), which could cause significant friction between the hydrogel and the nearby tissues to harm the loaded cells. On the contrary, we find that the MeHA@J@DM group presented significantly lowered COF due to the presence of the hydration p(DMA-MPC) layer (Figures 2G,H). We speculated that such drastically decreased COF could effectively mimic the lubrication microenvironment of the natural cartilage to enhance the cell viability of the implanted PJAC (Yang L. et al., 2021). In addition to the lubrication, the mechanical property of the hydrogel is also important. If the material is too soft, it cannot bear the external forces and will collapse after the implementation (Wu et al., 2022). Thus, we tested the mechanical properties of different groups with mechanical tester and found that the naked HA group presented lowest mechanical properties with Young’s modulus at ∼ 12.3 kPa (Figures 2H–J). On the contrary, the MeHA@J and MeHA@J@DM groups demonstrated nearly two folds of the Young’s modulus. This is because the minced PJAC could work as an enhancement filler to increase the mechanical properties of the hydrogels. Altogether, we demonstrated that the MeHA@J@DM presented excellent lubrication properties and enhanced mechanical properties which holds great potential for cartilage regeneration.

**FIGURE 3:**
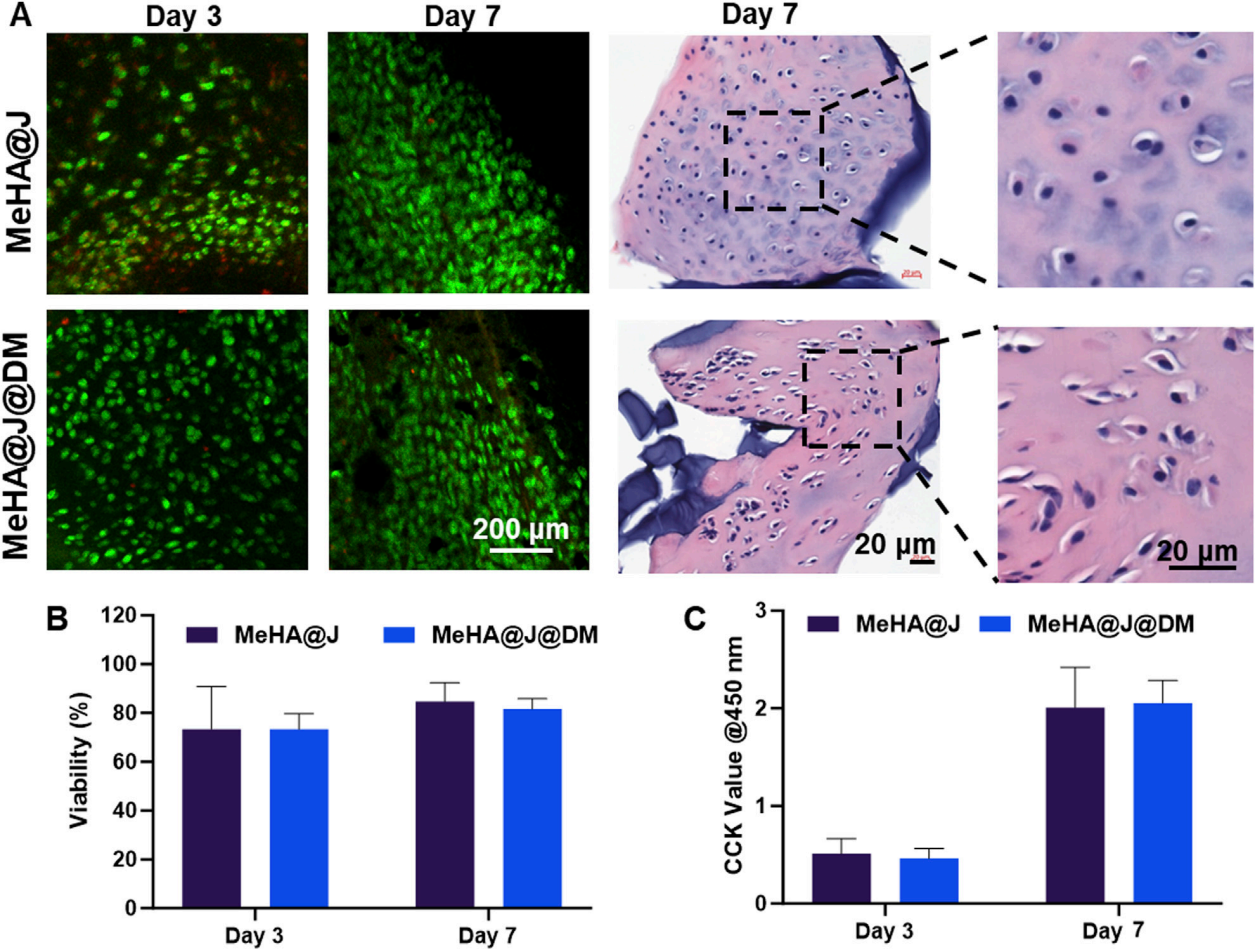
Biocompatibily of MeHA@J and MeHA@J@DM. **(A)** Live/Dead and H&E staining of the cultured MeHA@J and MeHA@J@DM; **(B,C)** Cell viability and proliferation of different hydrogels.

During the PJAC implantation, chondrocyte viability is one of the core concerns for the successful implementation (Lanham et al., 2017). Therefore, we evaluated the cell viability of the MeHA@J and MeHA@J@DM groups by live and dead staining on day 3 and day 7. We find that both groups presented over 70% cell viability on day 3 and day 7 (Figures 3A,B). The results on day 1 and day 14 indicated similar trends (Supplementary Figure S3). Under H&E staining, we also observed that the cultured cartilage fragments still exhibit dense clusters of chondrocytes, indicating that the MeHA and DM coating did not lead to degradation of cartilage *in vitro* (Figure 3A). Such tremendous cell viability could result from the excellent protective effect of the HA. We also evaluated the cell proliferation of the PJAC loaded in the hydrogels. We found that both MeHA@J and MeHA@J@DM groups presented good cell proliferation in day 3 and day 7 (Figure 3C). Above results indicated the good cell biocompatibility of our proposed hydrogel systems.

**FIGURE 4:**
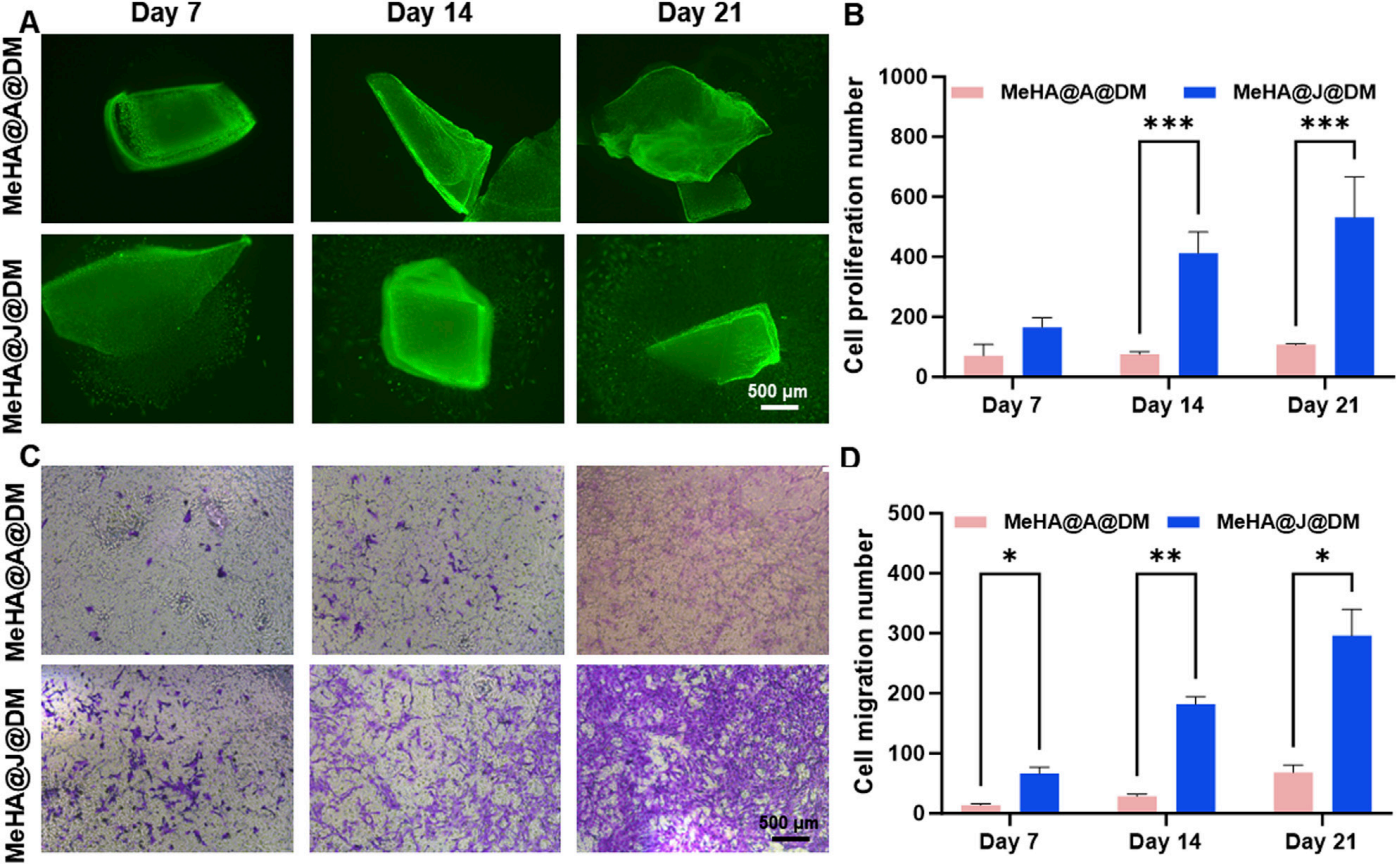
Cell migration and proliferation in MeHA@J@DM hydrogel compared to MeHA@A@DM. **(A)** Representative images showing migrated cartilage cells in MeHA@J@DM and MeHA@A@DM on day 7 of *in vitro* culture. **(B)** Quantification of cell proliferation in MeHA@J@DM and MeHA@J@DM. **(C)** Violet staining of cartilage cells in transwell flask culture of MeHA@J@DM and MeHA@A@DM hydrogels. **(D)** Quantification of the migration cell number. A difference at *p < 0.05, **p < 0.01 and ***p < 0.001 was considered statistically significant.

We further performed additional long-term *in vitro* culture experiments to investigate the potential of the MeHA@DM hydrogel in preserving the regenerative properties of PJAC. To validate the efficacy of our MeHA@DM hydrogel, we included a control group consisting of particulated adult articular cartilage (PAAC) embedded in a hydrogel formulation referred to as MeHA@A@DM. The PAAC group served as a comparison due to its inherently lower proliferation ability compared to PJAC when encapsulated in the MeHA@A@DM hydrogel (Adkisson IV et al., 2010). This control group was utilized to further substantiate the regenerative benefits conferred by our MeHA@DM hydrogel. On day 7, we observed migrated cartilage cells in the MeHA@J@DM group, while the MeHA@A@DM group showed a scarcity of cells migrated from the cartilage (Figure 4A). This observation can be attributed to the higher bioactivity exhibited by PJAC compared to PAAC, as well as the effective preservation of bioactivity facilitated by the MeHA@DM hydrogel. Throughout the extended period of *in vitro* culture, both MeHA@J@DM and MeHA@A@DM hydrogel formulations supported the migration and proliferation of cartilage cells. This demonstrates the exceptional performance of our MeHA@DM hydrogel in facilitating the delivery of living materials. As the cell culture time extended, the MeHA@J@DM group exhibited a higher number of proliferating and migrating cells compared to MeHA@A@DM (Figure 4B). There was no significant difference in the results between MeHA@J@DM and MeHA@J (Supplementary Figure S4). These results were consistent when the cultures were performed in transwell flasks, with the MeHA@J@DM group showing increased cell transfer in the vertical transwell flask (Figures 4C,D). These findings indicate that our MeHA@DM hydrogel can effectively protect the viability of live materials in PJAC and support the migration of cartilage cells. These findings strongly indicated that the MeHA@J@DM hydrogel hold promising potential for augmenting the regenerative capacity of PJAC. By effectively preserving viable cells and facilitating their migration both within and beyond the hydrogel matrix, our MeHA@J@DM hydrogel demonstrates a favorable environment for enhanced cartilage regeneration (Lei et al., 2021).

**FIGURE 5:**
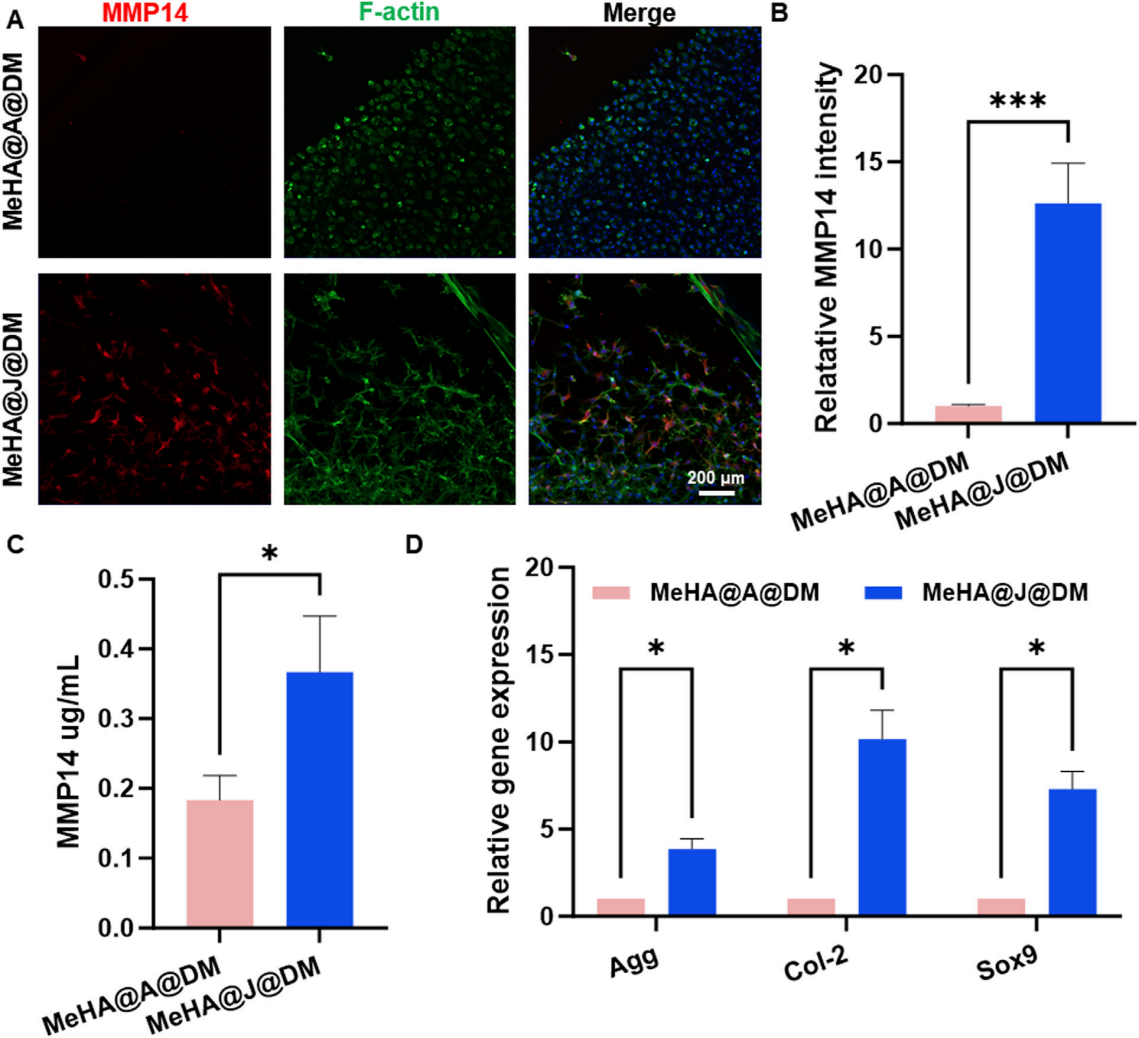
MMP-14 expression and regenerative potential in MeHA@J@DM and MeHA@A@DM. **(A)** Immunofluorescence staining showing MMP-14 expression in both cartilage and migrated cartilage cells in MeHA@J@DM and MeHA@A@DM on day 14 of culture. **(B)** Quantification of MMP14 expression. **(C)** ELISA analysis of the culture medium of MMP14 secretion in MeHA@J@DM and MeHA@A@DM. **(D)** PCR results of cartilage cells derived from MeHA@J@DM and MeHA@A@DM. A difference at *p < 0.05 and ***p < 0.001 was considered statistically significant.

MMP-14 is known to play a significant role in cartilage remodeling and regeneration (Mazor et al., 2014). It is involved in the turnover and remodeling of the extracellular matrix during the growth and maturation of juvenile articular cartilage, ensuring proper formation and maintenance of the tissue (Liu et al., 2013). To further investigate the involvement of MMP-14, we compared its expression in MeHA@J@DM and MeHA@A@DM. Immunofluorescence staining of MMP-14 on day 14 revealed that MeHA@J@DM exhibited MMP14 expression in both the cartilage and migrated cartilage cells (Figure 5A). Notably, MeHA@J@DM demonstrated a 13-fold higher MMP-14 expression compared to the MeHA@A@DM group (Figure 5B). These findings were consistent with the results obtained from ELISA analysis of the culture medium, which showed that MeHA@J@DM released higher levels of MMP-14 compared to the MeHA@A@DM group (Figure 5C). The PCR results indicated that cartilage cells derived from MeHA@J@DM exhibited greater cartilage regenerative potential than those from MeHA@A@DM (Figure 5D). These results suggest that while MeHA@J@DM promotes MMP-14 expression and secretion, potentially facilitating cartilage remodeling and migration of cells.

**FIGURE 6:**
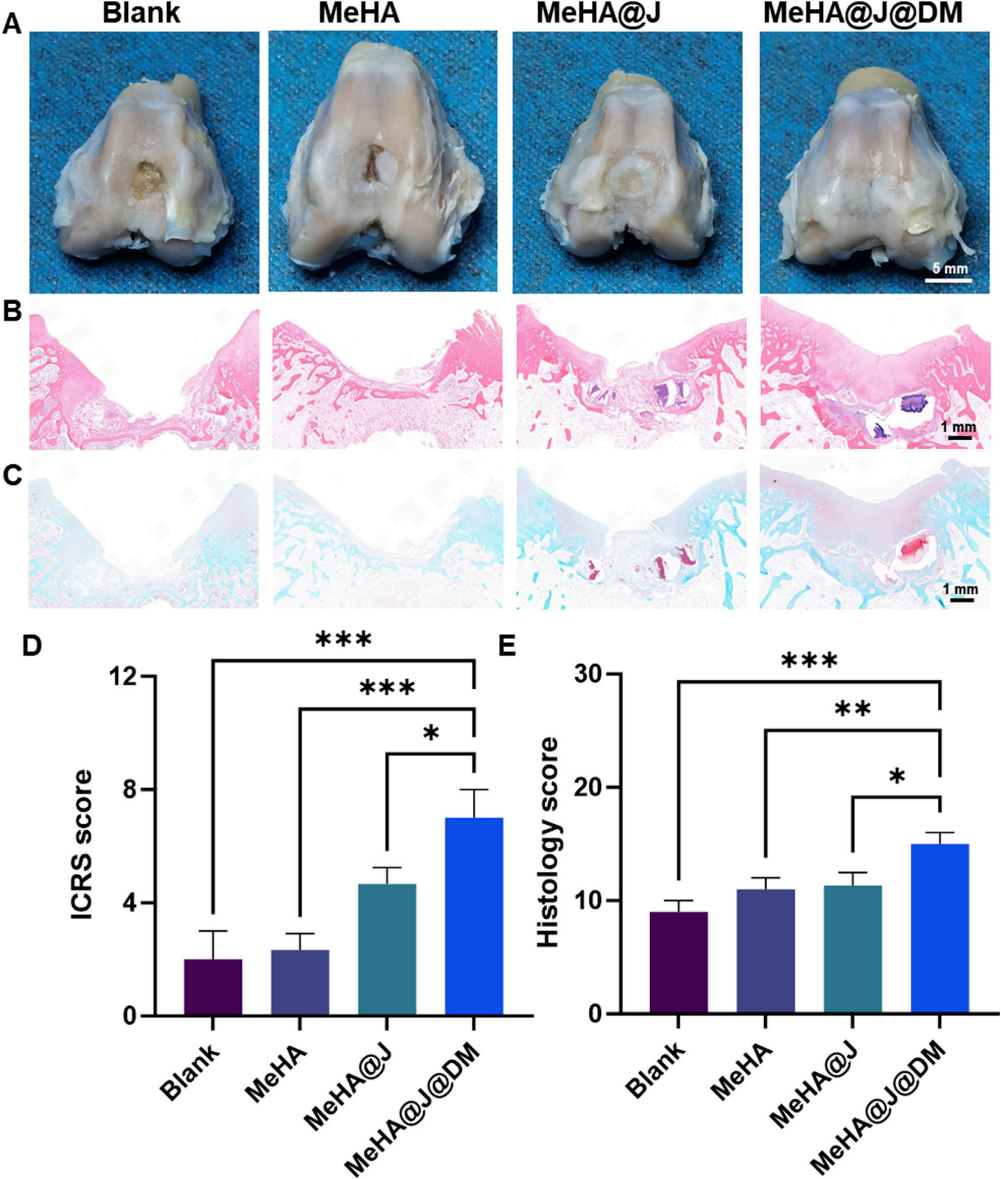
Histology analysis showing the *in vivo* cartilage regeneration efficacy of hydrogels in a rabbit knee cartilage defect model. **(A)** Macroscopical photographs of the repaired cartilage; **(B,C)** The HE and Safranin-O/Fast-Green staining of the repaired cartilage tissues. **(D,E)** ICRS and histology score of the regeneration cartilage and staining results. A difference at *p < 0.05 and ***p < 0.001 was considered statistically significant.

**FIGURE 7:**
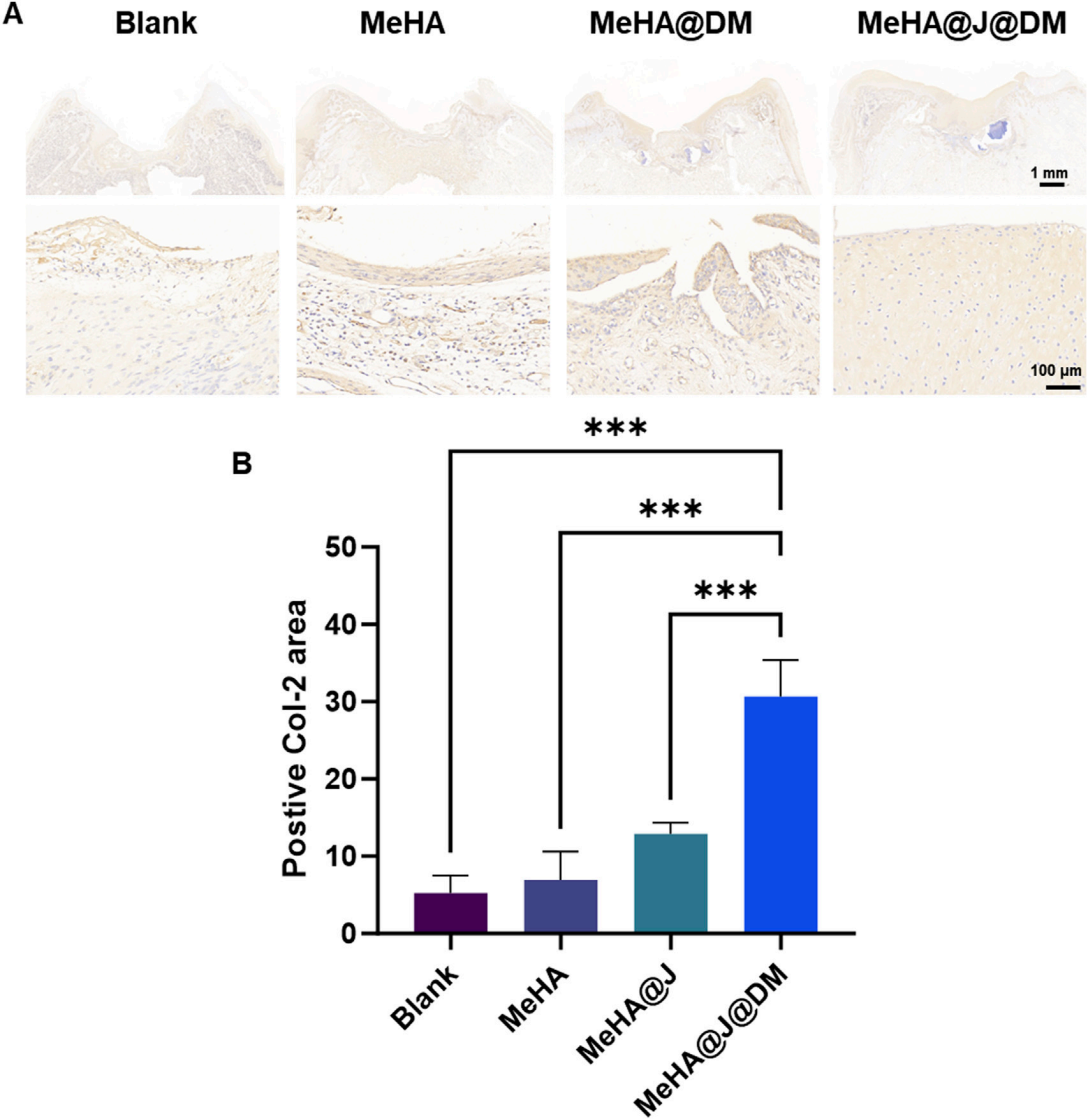
Histology analysis showing the *in vivo* cartilage regeneration efficacy of different hydrogels in a rabbit knee cartilage defect model. **(A)** Col-2 immunostaining of the regenerative cartilage. **(B)** Quantification of the Col-2 staining. A difference at *p < 0.05 and ***p < 0.001 was considered statistically significant.

While previous characterizations have demonstrated the promising *in vitro* cartilage regeneration efficacy of our MeHA@J@DM system, its long-term clinical relevance under physiological conditions remains unexplored. To address this, we conducted *in vivo* characterization of the therapeutic performance of the MeHA@J@DM carrier using a rabbit cartilage defect model (Supplementary Figure S5). The rabbits were divided into four groups: blank, MeHA, MeHA@J and MeHA@J@DM. At 12 weeks post-surgery, macroscopic photographs were taken to evaluate cartilage regeneration (Figure 6A). MeHA@J@DM exhibited the highest ICRS scores compared to the other groups, with 1.49-fold higher than MeHA@J (Figure 6D). These results indicated superior cartilage regeneration are attributed to the excellent bioactivity of PJAC and the water-retaining and lubricating properties of the DM coating layer. Histological staining was performed to further assess the tissue repair. In the blank and MeHA groups, minimal neo-cartilage tissue formation was observed at the implantation sites (Figure 6B). This could be attributed to the lack of protection from the pro-inflammatory microenvironment or inevitable friction in these groups. In contrast, the MeHA@J@DM group exhibited complete repair of the defect site, with tissue resembling native cartilage and excellent integration with the implant. To validate cartilage regeneration, we evaluated chondrogenesis through H&E and Safranin-O/Fast-Green staining (Figures 6B,C). MeHA@J@DM group demonstrated superior cartilage repair, with the defect sites filled with newly developed cartilage tissues rich in glycosaminoglycans (GAGs) (Figure 6C). Furthermore, H&E staining analysis with histological scores revealed that the MeHA@J@DM group achieved the highest scores among all the groups (Figure 6E). Collagen-2 (Col-2) is a key marker for cartilage repair, reflecting the enhanced production and deposition of cartilage extracellular matrix components (Roberts et al., 2009). The upregulation of Col-2 expression indicates the promotion of cartilage regeneration and the restoration of its structural integrity. We also performed the Col-2 staining and found that MeHA@J@DM groups demonstrate the largest positive Col-2 staining areas compared to other groups (Figures 7A,B). These results collectively indicate that MeHA@J@DM holds potential for the treatment of cartilage repair, as it exhibited superior cartilage regeneration and integration in the rabbit cartilage defect model. Further investigations are necessary to expand our understanding of the long-term effects and clinical applicability of the MeHA@J@DM system.

There are still some limitations in this study. First of all, although the SEM image of the hydrogel was shown in the article, the structure of the hydrogel was not uniform enough due to the cartilage fragments, so the representation of the picture was not strong. Second, due to the composition of articular synovial fluid and the non-vascular and nerve-free nature of cartilage, hyaluronic acid and cartilage fragments have low immunogenicity, but the modification of hydrogel may enhance the immunogenicity of hydrogel and induce inflammation and other immune reactions. Therefore, additional anti-inflammation may be necessary. Third, limited by the experimental conditions, this study failed to set the fibrin glue group as the positive control group. Fourth, we used rabbits as experimental animals, but failed to use experimental animals whose joints were more similar to those of humans.

## 4 Conclusion

In conclusion, we have successfully formulated the MeHA@J@DM hydrogel carrier for the delivery of PJAC for cartilage regeneration. The synthesis of MeHA and p(DMA-MPC) was confirmed using ^1^H NMR and FTIR analyses. The MeHA solution exhibited excellent flowability and could be rapidly crosslinked into a hydrogel using UV light. The injectivity and crosslinking ability of MeHA were validated, and different hydrogel structures were successfully constructed. The composite material MeHA@J was prepared by mixing MeHA with PJAC and crosslinking with UV light, followed by the coating with p(DMA-MPC) to obtain MeHA@J@DM samples. The MeHA@J@DM carrier demonstrated improved lubrication efficiency with a significantly lowered coefficient of friction (COF) compared to MeHA. Furthermore, the mechanical properties of MeHA@J and MeHA@J@DM hydrogels were enhanced, making them suitable for cartilage regeneration. The cell viability and proliferation of chondrocytes within the hydrogels were evaluated, showing excellent biocompatibility. *In vivo* studies using a rabbit cartilage defect model demonstrated that MeHA@J@DM exhibited superior cartilage repair outcomes, with the defect sites filled with newly developed cartilage tissues rich in GAGs. The expression of MeHA@J@DM group and MeHA@J group *in vitro* is similar, including the number and efficiency of cell migration, but due to its lower COF, its performance within the joint is better MeHA@J. The reasons may include avoiding further wear of articular cartilage, reducing inflammatory reactions caused by friction, and increasing the willingness of experimental animals to move around. These findings highlight the potential of MeHA@J@DM as a promising hydrogel carrier for cartilage regeneration applications.

## Data Availability

The original contributions presented in the study are included in the article/Supplementary Material, further inquiries can be directed to the corresponding authors.
