# Supplementary material for: Multifunctional MeHA hydrogel for living materials delivery with enhanced cartilage regeneration
Source: Front Bioeng Biotechnol. 2025 May 30;13:1545773. doi: 10.3389/fbioe.2025.1545773 (PMC12162624; doi:10.3389/fbioe.2025.1545773)
Supplement: Supplementary file 1 [file DataSheet1.docx]

**Natural cartilage inspired carrier for living materials delivery with** **enhanced cartilage regeneration**

Qunchao Chen^a,b1^, Lang Bai^b1^, Guoyang Wan^b1^, Yuefeng Hao^b^*, Xing Yang^b^*, Hongtao Zhang^a^*

^a^Department of Orthopedics, The Fourth Affiliated Hospital of Soochow University, Suzhou Dushu Lake Hospital, Medical Centre of Soochow University, Suzhou 215006, Jiangsu Province, China

^b^Orthopedics and Sports Medicine Center, The Affiliated Suzhou Hospital of Nanjing Medical University, Suzhou Municipal Hospital, Gusu School, Nanjing Medical University, 242 Guangji Road, Suzhou 215008, P. R. China.

*To whom correspondence may be addressed

E-mail: haoyuefeng@njmu.edu.cn (Y. H.), xingyangsz@njmu.edu.cn (X. Y.), htzhangsz@163.com (H. Z.)


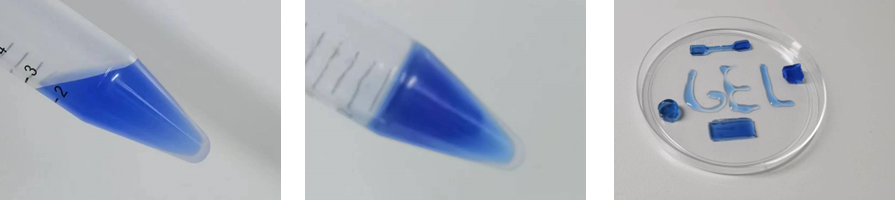


**Figure S1.** Crosslinking and injectability of MeHA hydrogel


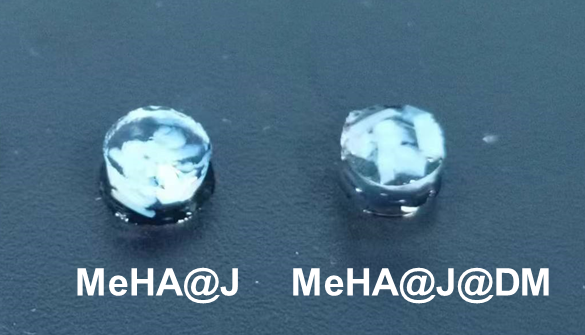


**Figure S2.** The macroscopic images of the MeHA@J and MeHA@J@DM hydrogels.

**
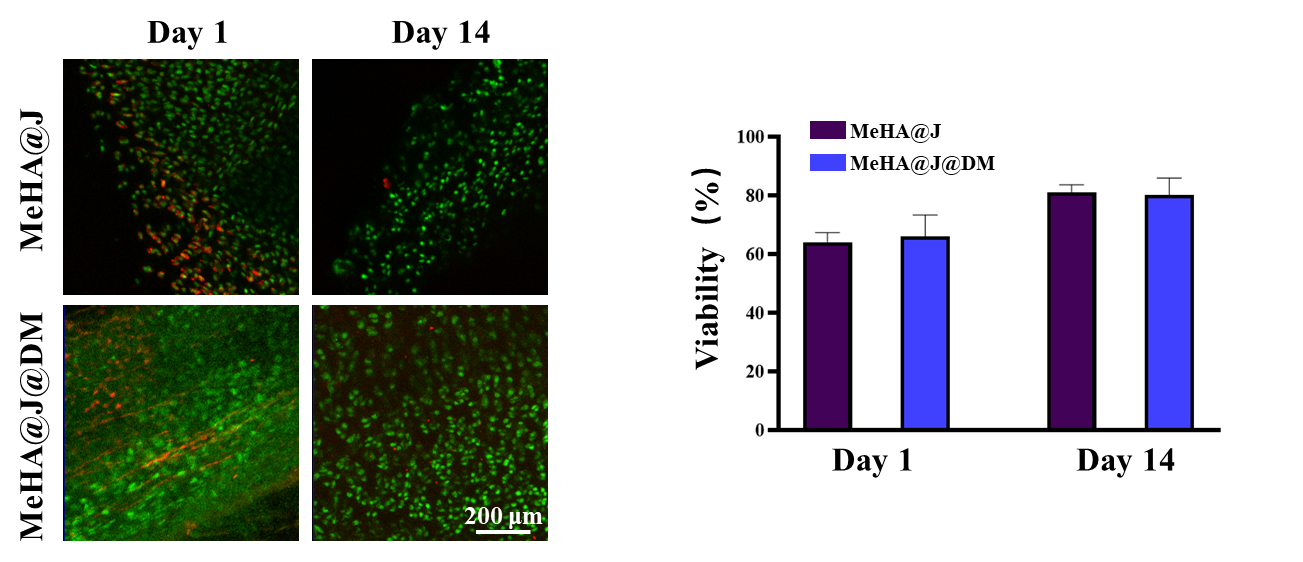
** **Figure S3.** Live/Dead staining of the cultured MeHA@J and MeHA@J@DM on day 1 and 14, and cell viability of different hydrogels.


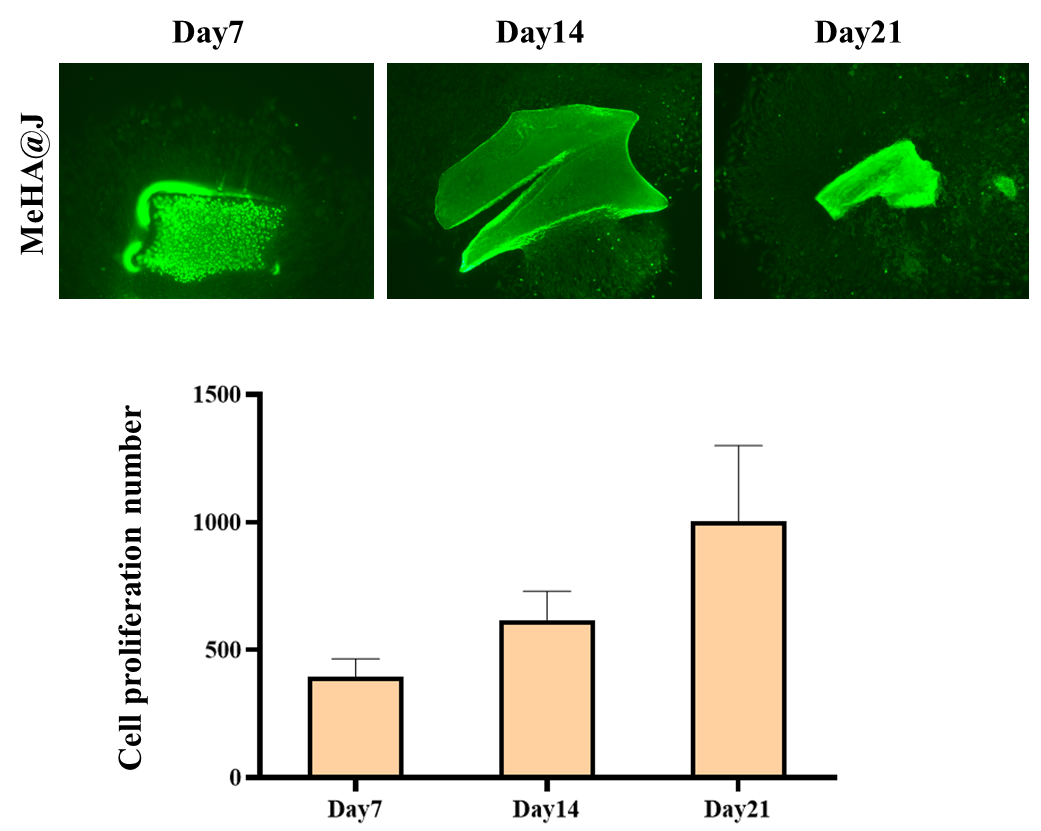


**Figure S4.** Representative images showing migrated cartilage cells in MeHA@J on day 7 of in vitro culture and quantification of cell proliferation.


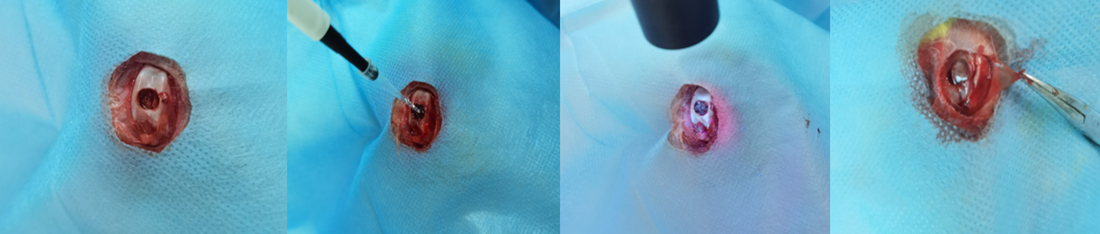


**Figure S5.** The process of implanting hydrogels in rabbit cartilage models.
